# Supplementary material for: Life expectancy drop in 2020. Estimates based on Human Mortality Database
Source: PLoS One. 2022 Jan 31;17(1):e0262846. doi: 10.1371/journal.pone.0262846 (PMC8803147; doi:10.1371/journal.pone.0262846)
Supplement: Attachment — Submitted filename: Response_to_reviewers.pdf [file pone.0262846.s001.pdf]

Manuscript PONE-D-21-15829, “Life expectancy drop in 2020. Estimates from Human Mortality Database”

We thank the reviewers for their comments, which we have tried to address. In essence, reviewers asked to revise the paper in terms of the quality of English language. A detailed description of our responses to reviewers’ concerns follows:

- **Reviewer 1**: The concept and result are well presented, but with some errors in standard English usage that might cause some ambiguity and should be addressed.

Thanks for raising this point. The manuscript has been thoroughly revised by a professional language editing service to improve the grammar and readability.

- **Reviewer 2**: In the abstract, it says the US life expectancy dropped by 1.69 years and 1.83 in Spain. These do not correspond with the data in the manuscript. Please take a look at this discrepancy and correct either the abstract or the results and table.

We apology for this, we missed to update the figures in the abstract, now this has been corrected.

- **Reviewer 2**: Minor grammatical issues:
Lines 3-5: This sentence is unclear and awkward. Please revise.
Line 79: "deacline" should be "decline"
Line 106: Replace "diffusion" with "spread"

Thanks for raising this point. The manuscript has been thoroughly revised by a professional language editing service to improve the grammar and readability.
