# Supplementary material for: Life expectancy drop in 2020. Estimates based on Human Mortality Database
Source: PLoS One. 2022 Jan 31;17(1):e0262846. doi: 10.1371/journal.pone.0262846 (PMC8803147; doi:10.1371/journal.pone.0262846)
Supplement: Attachment — Submitted filename: Response_to_reviewers__2.pdf [file pone.0262846.s002.pdf]

## Manuscript PONE-D-21-15829, “Life expectancy drop in 2020. Estimates from Human Mortality Database”

We thank the reviewer for her/his further comments, which we have tried to address. It should be noted that in the meantime the mortality database has been updated, so we re-run our analyses and the figures have changed a bit. Since now data are available also for Russia, this country has been included in the analysis (and it turns out that it is the one with the highest Life expectancy drop).

As for the points raised by the reviewer:

• In the first line of the introduction, it is mentioned: “after one year”. It has been more than a year since the start of the pandemic. Please update.

*Agreed, we dropped it.*

**• The exact definition of “Life expectancy at birth” should be stated in the introduction. Also, what is the difference between this factor and “life expectancy”.**

*A definition of life expectancy (and life expectancy at birth) has been included with relative quotation.*

**• The discussion needs to cover some similar studies to compare. This part could have been written more comprehensively to better highlight and interpret the results in the context of previous research. Also, mention the limitations and strengths of the present study.**

*We included a separate discussion section presenting strengths and limitations of using life expectancy drop as a measure of the pandemic impact, in comparison with other measures (covid-19 mortality, excess of death).*

**• It is better to include a separate conclusion paragraph.**

*Agreed, we included a separate discussion and conclusion sections.*

**• There are still some grammatical and dictation issues (e.g. line 120: WhileIn contrast, line 60: xhich is)**

*Thanks for spotting these, we fixed them and edited the whole manuscript*

**• The article can also be considered as a short communication, based on the editor’s decision.**

*Thanks, if it fits with the journal editorial rules, we are supportive of your suggestion.*
